# Inorganic Polyphosphate in Hematolymphoid Malignancies: Biological Rationale and Emerging Research Gaps

**DOI:** 10.3390/biom16071036

**Published:** 2026-07-16

**Authors:** Francisco García-Domínguez, Cecilia Fernandez-Ponce, Carlota Salb-Pernas, Antonio Santisteban-Espejo, Felix A. Ruiz

**Affiliations:** 1 Clinical Management Unit in Pathology, Puerta del Mar University Hospital, 11009 Cadiz, Spain; francisco.garcia.dominguez.sspa@juntadeandalucia.es; 2 Clinical and Translational Research in Hematology Group (EM-34), Biomedical Research and Innovation Institute of Cadiz (INiBICA), Puerta del Mar University Hospital, 11009 Cadiz, Spain; ceciliamatilde.fernandez@uca.es (C.F.-P.); carlota.salbpernas@alum.uca.es (C.S.-P.); 3 Department of Biomedicine, Biotechnology and Public Health, Faculty of Medicine, University of Cadiz, 11003 Cadiz, Spain; 4 Department of Medicine and Surgery, Faculty of Medicine, University of Cadiz, 11003 Cadiz, Spain

**Keywords:** polyphosphate, hemostasis, inflammation, myeloma, leukemia, lymphoma

## Abstract

Inorganic polyphosphates (polyP) are linear phosphate polymers that play essential functions in human physiology, such as coagulation and inflammation. Despite their recognized participation in solid tumor progression, there are few data on hematolymphoid malignancies. Here, we conducted a narrative review of studies investigating polyP functions in mammalian biology, coagulation, and hematologic systems, focusing on research gaps in hematological oncology. PolyP modulates coagulation cascades, platelet activation, and cellular metabolism. In tumors, polyP has been reported to modulate immune responses and apoptosis in several experimental systems. Emerging data suggest participation in leukemic signaling and the bone marrow microenvironment. Direct evidence linking polyP to hematolymphoid neoplasms is scarce. Being biologically plausible, it represents a promising and largely unexplored area of research and an excellent opportunity for novel therapeutic approaches.

## 1. Introduction

In this review, we aim to summarize the biological functions of polyP in human physiology, evaluate its established roles in general cancer biology, examine the current evidence in hematolymphoid pathology, and identify the critical gaps and future directions for research in this field. A narrative literature search was conducted according to a strategy described in detail in the Supplementary Information.

### 1.1. Polyphosphate, a Versatile Polymer

**Figure 1:**
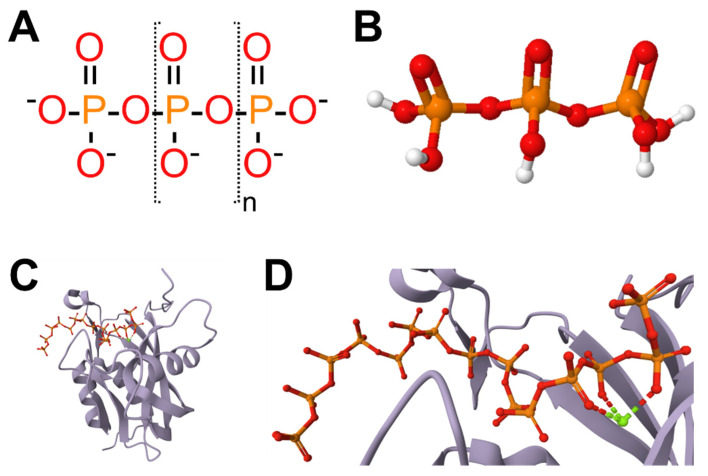
Basic structure of polyP. (**A**) Schematic representation of linear polyP, with its atoms in a CPK color scheme, consisting of n repeating orthophosphate units (P_i_, between brackets), connected by high-energy phosphoanhydride bonds. PolyP moieties can have from 3 to hundreds of P_i_ units. (**B**) Three-dimensional representation of tripolyphosphate. The image was made with the web-based tool “DIY-molecules”, setting a spatial optimization of 300 steps [9]. (**C**,**D**) PolyP is versatile, adopting different spatial conformations when it interacts with proteins. Shown here is an example of a protein structure (DDP1) bound to a 15-unit polyP, determined by X-ray diffraction [3]. Panel (**C**) shows the complete DDP1 protein structure, and Panel (**D**) shows a close-up of the polyP. The structures were visualized, and the images were downloaded, using the Mol* 3D Viewer program from the RCSB PDB [10].

Inorganic polyphosphates (polyP) are molecules consisting of linear chains of phosphate residues (P_i_) linked by high-energy phosphoanhydride bonds (Figure 1A,B) [1]. The tetrahedral conformation of the P_i_ units and relative flexibility of the phosphoanhydride bonds allow the polyP to adopt different spatial conformations [2]. This gives it broad versatility and enables it to interact with several proteins and other biomolecules. Figure 1C,D show an example of a crystal structure protein bound to a 15-unit polyP determined by X-ray diffraction: diadenosine and diphosphoinositol polyphosphate phosphohydrolase (known as DDP1 in yeast or DIPP1 in humans) [3].

PolyP are recognized as main regulators in mammalian systems, although they were once considered primitive molecules. In humans, polyP is primarily stored in the dense granules of platelets and released upon activation, where it exerts profound biological effects, particularly in coagulation and systemic inflammation [4,5].

Over the past two decades, the relevance of polyP in human biology has grown considerably. It was initially recognized for its foundational role in hemostasis, especially its ability to integrate the clotting cascade and innate immunity, and now, it is understood to participate in a diverse array of processes. These include complement system activation, immune modulation, and cellular signaling pathways [6,7,8]. These multifaceted functions position polyP at a critical intersection of thrombosis, inflammation, and cancer biology.

### 1.2. Mammalian PolyP Metabolism

The mechanisms of polyP synthesis and degradation have been comprehensively reviewed by our group and others [1,11,12,13,14,15]. The overall message conveyed in these reviews is that the enzymes involved in polyP synthesis and degradation have been well characterized in prokaryotes and unicellular eukaryotes. However, in higher eukaryotes, including mammals, there are no homologs of the enzymes described in lower organisms, and they are only just beginning to be elucidated. A list of the enzymes proposed as involved in polyP metabolism in mammals is shown in Table 1.

### 1.3. Subcellular Localization of polyP in Mammalian Cells

PolyP appears to be present in all the cells studied; however, its localization varies by cell type. This highly compartmentalized distribution suggests that its biological functions are strongly dependent on subcellular localizations and local concentrations [18,19,20]. A summary of the subcellular localizations observed for polyP in mammals is provided in Table 2.

## 2. Polyphosphates in the Hematological System

### 2.1. Role in Hemostasis and Platelet Biology

PolyP is a fundamental regulator of the coagulation system. It accelerates the thrombin-mediated activation of Factor V, thereby enhancing thrombin generation, and significantly stabilizes the structure of fibrin clots, making them more resistant to premature degradation [4,5,7]. Factor V is a cofactor that, once activated to FVa, works with Factor Xa to form the prothrombinase complex, which catalyzes the rapid conversion of prothrombin to thrombin. In addition, polyP accelerates thrombin-mediated activation of factor XI [4]. This creates a feedback loop within the coagulation system, leading to a massive burst of thrombin generation from a small amount of factor XIa, which in turn comes from a smaller amount of thrombin. Another function of polyP in thrombin generation is triggering the contact pathway. PolyP triggers the contact pathway by promoting the factor XII activation, which serves as the starting point for clotting [1,4]. Its anionic surface provides a scaffold for the autoactivation of factor XII, further driving the cascade toward thrombin production. Finally, once generated, thrombin converts fibrinogen into fibrin, a process modulated by polyP through its interaction with the developing network to enhance the structure of fibrin fibrils and make it more resistant to fibrinolysis, allowing for effective wound healing [4,7].

Platelet-derived polyP (typically 60–100 residues in length) acts as a potent procoagulant mediator, linking thrombosis and acute inflammation [7]. Extracellular polyP acts as a chemotactic agent which attracts neutrophils to the site of platelet activation or injury, promoting the formation of Neutrophil Extracellular Traps (NETs) [33,34]. PolyP also promotes the differentiation of monocytes into macrophages and fibrocytes, modulates the complement system, and activates diverse signaling pathways (such as the ERK1/2-EGR1 signaling axis), which help reprogram cellular responses during inflammation [4,7] and are essential to the regulation of hemostasis.

Overall, polyP can be regarded as a “cellular Swiss Army knife”: predominantly secreted by platelets, it ensures an efficient and sustained hemostatic response while also modulating inflammation—a process in which it plays a central role [35].

### 2.2. Role in Inflammation and Immunity

As described above, polyP interacts with various components of the immune system. It is a potent modulator of the complement system, crucial for the innate immune response. This is important for the clearance of pathogens and damaged cells and suggests that these molecules could act as a “damage-associated molecular pattern” when released in excess by both platelets and mast cells [1,2,7].

PolyP activates several intracellular pathways:

- ERK1/2-EGR1 Axis: When polyP is produced or released, it can trigger the ERK1/2-EGR1 signaling pathway, which reprograms the cell transcriptome and proteome [8].
- Ras and Akt Signaling: In mammalian cells transfected with a yeast polyphosphatase it was demonstrated that polyP can influence cell development and survival through Ras and Akt proteins [36].
- Astrocyte Signaling: In the brain, polyP acts as a gliotransmitter, transmitting signals between astrocytes by activating P2Y1 receptors and stimulating phospholipase C [1].

## 3. Polyphosphates in Cancer Biology

### 3.1. Tumor Growth, Survival, and Metabolic Adaptation

In oncology, polyP has been implicated in promoting of tumor cell proliferation and survival. Cancer cells have exceptionally high energy requirements to maintain rapid growth, and polyP serves as a critical energy-rich reservoir in certain experimental models [37]. PolyP is highly abundant in breast, lung, prostate and brain cancer, being “avidly consumed” by cancer cells during periods of glucose deprivation to maintain metabolic functions. This leads to a polyP depletion, which reduces the tumor cell’s ATP stores [37]. This suggests that polyP acts as energy source. Also, myeloma plasma cells contain significantly higher levels of intracellular polyP than normal plasma cells or other B-cell populations from healthy donors [29]. In this case, intracellular polyP in myeloma cells is concentrated within the nucleolus, colocalizing with nucleolar markers like fibrillarin and B23. This points to a functional role as a dose-dependent modulator of RNA polymerase I activity, which is necessary for their continuous proliferation [29,38].

These effects are often mediated through the modulation of intracellular signaling pathways, such as the mTOR (mammalian target of rapamycin) pathway [39]. This central kinase coordinates cell growth and protein synthesis in response to nutrient availability. In mammary cancer, the removal of polyP leads to failure of mTOR activation, resulting in severely reduced growth even in the presence of mitogens like insulin.

Furthermore, polyP may influence mitochondrial function and cellular energy metabolism, providing cancer cells with a competitive advantage in nutrient-deprived environments [36]. The levels of mitochondrial polyP are closely tied to the cell’s energy state, since the inhibition of mitochondrial ATP synthase significantly decreases polyP levels and depleting mitochondrial polyP drastically reduces ATP stores and impairs viability [37]. Interestingly, while internal polyP supports survival, external polyP treatment can disrupt mitochondrial energy metabolism, reducing cellular ATP levels and making cells more sensitive to external stressors such as radiation [40]. PolyP also regulates the Permeability Transition Pore (PTP). This is a mitochondrial membrane protein complex that, when open, causes mitochondria to lose their membrane potential and leads to cell death [37]. The effect of polyP on PTP depends on the length of the inorganic polymer. Long-chain polyP forces PTP to open and causes cell death, while short and medium-length polyP chains are involved in normal signal transduction and metabolism [1].

### 3.2. Angiogenic Microenvironment

PolyP is a key contributor to the “angiogenic switch.” It regulates endothelial cells behavior and enhances the activity of various growth factors, including basic fibroblast growth factor (bFGF) [7]. PolyP blocks the activity of bFGF, producing an anti-angiogenic effect by binding bFGF to its cognate cell-surface receptors. By blocking bFGF, polyP inhibits the activation of critical downstream signaling pathways in human endothelial cells, specifically ERK (extracellular signal-regulated kinase) and p38 MAPK (mitogen-activated protein kinase). Because neovascularization is essential for tumor spread, the intravenous administration of polyP has been shown to suppress pulmonary metastasis in an experimental mouse model B16BL6 melanoma cells by preventing the formation of new microvessels within and around metastasized colonies [14].

### 3.3. Immune Evasion and Metastatic Spread

Emerging evidence suggests that polyP can modulate the immune response within the tumor microenvironment, potentially facilitating tumor cell immune evasion. High concentrations of extracellular polyP have been shown to inhibit the proliferation of leukocytes by inhibiting proteasome activity in peripheral blood mononuclear cells by approximately 55% at 150 µM—an effect mechanistically conserved from *Dictyostelium* to human leukocytes [7]. Additionally, extracellular polyphosphate acts as a concentration-dependent bidirectional chemotactic signal for neutrophils, inducing chemoattraction at gradient concentrations of 0–10 pM and chemorepulsion at 0–100 pM [7]. This suppression of leukocyte proliferation could limit the body’s ability to mount an effective anti-tumor immune response [14]. In the context of hematological malignancies, the net effect of sustained high polyP in the tumor microenvironment may include suppression of cytotoxic lymphocyte expansion, thereby favoring immune evasion rather than tumor elimination [7].

### 3.4. Metastatic Spread

By altering the physical properties of the extracellular matrix and interacting with circulating platelets, polyP also facilitates the survival of circulating tumor cells (CTCs) during the metastatic journey [3]. Intracellular polyP acts as an energetic reserve that cancer cells “avidly consume” during starvation to maintain metabolic stability and fuel processes like proliferation, migration, and the epithelial–mesenchymal transition. The human polyphosphatase h-prune (which degrades polyP) is also implicated in cell migration and metastasis formation, suggesting that the precise regulation of polyP levels is critical for a tumor’s invasive capacity [14].

Additionally, polyP affects the tumor–stroma interaction, creating a microenvironment that facilitates tumor expansion and eventual metastatic spread. The tumor microenvironment often contains serum P_i_ concentrations that are two to four times higher than those in healthy individuals [7]. Ecto-enzymes in the microenvironment release this P_i_, which is then internalized by specialized transporters to fuel proliferation, migration, and the epithelial–mesenchymal transition by producing reactive oxygen species [18].

## 4. Direct Evidence in Hematolymphoid Malignancies: PolyP in Multiple Myeloma

**Figure 2:**
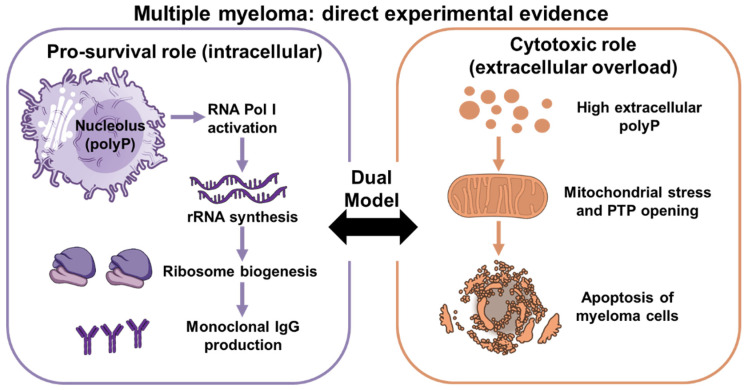
PolyP in hematolymphoid malignancies: Multiple myeloma: direct experimental evidence. PolyP plays a dual role: Intracellular polyP, concentrated primarily in the nucleolus, drives rRNA synthesis and may potentially support the production of monoclonal immunoglobulins [16]. Extracellular polyP at high concentrations induces apoptosis in myeloma cells. All illustrations used were public-domain resources from NIAID NIH BioArt Source (https://bioart.niaid.nih.gov/ accessed on 1 April 2026).

Among all hematological malignancies, multiple myeloma is the only entity for which direct experimental data on polyP biology currently exist (Figure 2). In Hernandez-Ruiz et al. [38], we demonstrated that exogenous polyP induces apoptosis selectively in human plasma cells from myeloma patients, with significantly less effect on non-malignant lymphoid populations. The mechanism may involve mitochondrial membrane depolarisation, consistent with the established role of long-chain polyP in forcing the opening of the mitochondrial PTP [1,5]. This selectivity for malignant plasma cells may reflect their elevated baseline mitochondrial stress, their dependence on oxidative phosphorylation, or their distinct intracellular polyP content. Complementing this finding, in Jimenez-Nunez et al. [16], we demonstrated that myeloma cells contain significantly higher levels of intracellular polyP than normal plasma cells or other B-cell populations from healthy donors, with this polyP concentrated within the nucleolus. Nucleolar polyP in myeloma cells was shown to function as a dose-dependent modulator of RNA polymerase I activity, thereby directly supporting ribosomal RNA synthesis and the biosynthetic machinery that sustains the high-rate production of monoclonal immunoglobulin [16]. These two studies together define an internally consistent picture: myeloma cells accumulate polyP to fuel ribosomal transcription and protein synthesis; however, when intracellular polyP levels are perturbed or extracellular polyP exceeds a threshold concentration, the underlying mitochondrial sensitivity of these metabolically stressed cells renders them vulnerable to PTP-driven apoptosis.

This duality could be exploited therapeutically if the relevant concentration ranges and delivery mechanisms are established in vivo. Beyond these direct observations, the biology of myeloma intersects with polyP in at least three additional ways that have not yet been experimentally addressed and which we propose here as research hypotheses. Hypothesis 1: Myeloma’s profound dependence on the bone marrow microenvironment—mediated by adhesion molecules, growth factors, and vascular niches—overlaps extensively with the domains in which polyP has documented activity, including endothelial regulation and extracellular matrix signaling [36]; whether polyP actively contributes to niche remodeling in this disease remains untested. Hypothesis 2: The prothrombotic phenotype of myeloma patients, substantially amplified by immunomodulatory drugs, is incompletely explained by conventional risk factors; polyP, as a major amplifier of the contact and common coagulation pathways [3,4], represents a biologically plausible and yet unstudied contributor. Hypothesis 3: Myeloma progression is driven in part by immune dysfunction within the marrow niche, and polyP’s capacity to suppress lymphocyte expansion [7] while promoting myeloid differentiation could theoretically contribute to the immunosuppressive environment that allows myeloma to escape immunological control (Figure 2).

## 5. Possible polyP Roles in Other Hematological Malignancies

**Figure 3:**
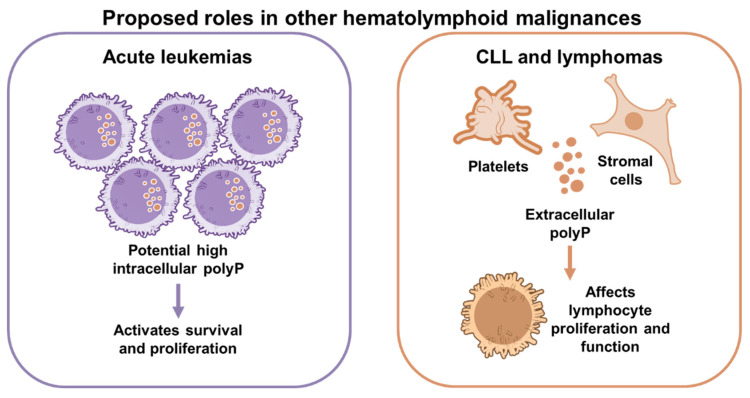
Proposed roles of polyP in other hematolymphoid malignancies (hypotheses and preliminary results). (**Left** panel) As in multiple myeloma and solid tumors, intracellular polyP may drive proliferation and survival in leukemias. (**Right** panel) Similarly, the polyP secreted into the extracellular space by platelets, stromal cells, and tumor cells themselves may modulate the microenvironment and lymphocyte populations. All illustrations used were public-domain resources from NIAID NIH BioArt Source (https://bioart.niaid.nih.gov/ accessed on 1 April 2026).

The hypotheses of the roles of polyP in leukemia and lymphoma are summarized in Figure 3. In acute leukemias, the accelerated proliferation of malignant blasts could plausibly require, and may be supported by, elevated intracellular polyP levels, given its established role as an energetic reserve and proliferation in cancer cells [11,36,39,41]. In chronic lymphocytic leukemia and lymphomas, where immune evasion is a dominant pathophysiological theme, the immunosuppressive effects of extracellular polyP on lymphocyte proliferation [8] suggest that polyP released by tumor-associated platelets or stromal cells may contribute to the exhaustion of anti-tumor immunity. Similarly, the role of h-prune (a human polyphosphatase whose overexpression correlates with metastatic potential [42]) has not been characterized in lymphoma or leukemia. This absence of data reflects a systematic failure to ask the question: whether polyP levels differ between malignant and normal hematopoietic populations across disease entities, and whether manipulating polyP in disease-relevant cell lines or primary patient samples produces measurable biological effects.

Preliminary observations from our laboratory suggest the presence of intracellular polyP in Hodgkin and Reed–Sternberg (HRS) cells from patients with classical Hodgkin Lymphoma (cHL; Salb, Santisteban-Espejo, and Ruiz, unpublished results). However, these findings remain unpublished and require independent validation and functional studies to determine whether polyP contributes to disease biology.

It has been reported that Hodgkin and HRS produce extracellular vesicles (EVs), which act as mediators of communication in the tumor microenvironment [43]. In clinical contexts, a correlation between high EV serum levels in cancer patients and an increased susceptibility to thrombotic events has been observed [44], which affects approximately 12% of cHL patients [45] due to the attached PolyP on the EV surface that activates the coagulation cascade [44]. PolyP may offer a new therapeutic target in this disease, since up to 10% of cHL patients do not respond to available treatments and die within a 5-year period [46].

For now, the most immediate experimental evidence in disease-relevant systems, requires: (i) quantification of polyP levels, intracellular and extracellular, across hematological malignancy subtypes, using patient-derived samples and standardized methodology; (ii) establishment of whether malignant hematopoietic cells from leukemia and lymphoma display the same polyP accumulation observed in myeloma [16]; (iii) functional studies in disease-specific cell lines and primary samples to determine the consequences of polyP depletion or supplementation on proliferation, survival, and signaling; and (iv) preclinical testing of polyP-targeted agents in hematological tumor models. Without this foundational work, the therapeutic proposals outlined above remain speculative, but biologically plausible.

A secondary priority is methodological. Current methods for analyzing polyP and the associated difficulties have been described recently by Schoeppe et al. [13]. PolyP quantification in biological matrices is technically demanding, and the lack of standardized assays contributes to variability across studies and limits cross-disease comparisons. Investment in validated, reproducible measurement platforms, whether fluorimetric, enzymatic, or mass spectrometric, would benefit the entire field and is a prerequisite for meaningful clinical biomarker studies.

## 6. Conclusions

In these two decades, polyP has opened a fascinating field of research that could lead to new perspectives on cancer, especially hematolymphoid tumors. While its role in hemostasis and inflammation has already been approached and well-described, studies on polyP and cancer are relatively new and have not been addressed in all kinds of tumors, such as leukemias or lymphomas. A summary of established findings, indirect evidence, and hypotheses presented in this review is provided in Table 3.

Further investigation is necessary, since it could open new therapeutic options in the future. Among others, some key questions in polyP and hematologic malignancies are: Do leukemic cells accumulate intracellular polyP? Does platelet-derived polyP influence leukemia progression? Is polyP a biomarker of bone marrow microenvironment remodeling? Can polyP modulation enhance immunotherapy responses?

## Figures and Tables

**Table 1:** Enzymes proposed as regulating polyphosphate metabolism in mammals.

Candidate Protein	Proposed Role in polyP Metabolism	Level of Evidence/References
F1-F0-ATP synthase	ATP synthase/proposed polyP synthase in mitochondria.	Indirect/Baev et al. [16]
DIPP1-3 (DIPP1 = Nudt3)	Endopolyphosphatases ^1^. Nudt3 mediates oxidative stress in response to polyP.	Strong/Lonetti et al. [17], Samper-Martin et al. [18], Kumble et al. [19].
h-Prune	Exopolyphosphatase ^1^. Associated with tumor progression.	Moderate/Tammenkoski et al. [20], Scoma et al. [21].
TRAP	Exopolyphosphatase ^1^. Related to bone resorption by osteoclasts.	Moderate/Harada et al. [22]
ALP (Alkaline phosphatase)	Exopolyphosphatase ^1^. Proposed as principal regulator of extracellular polyP.	Strong/Lorenz et al. [23]

^1^ Exopolyphosphatases cleave terminal P_i_ moieties from the polyP chain. Endopolyphosphatases cleave internal P_i_ bonds from the polyP chain. For polyP enzymes described in prokaryotes and unicellular eukaryotes [1,11,12,13].

**Table 2:** Subcellular localization and proposed mechanism of inorganic polyphosphate (polyP).

Cellular Localization	Proposed Molecular Mechanisms	References
Secretory granules (platelets, mast cells = acidocalcisome-like structures)	Release upon cell activation; contribution to coagulation pathways and bradykinin-mediated signaling	Ruiz et al. [24], Moreno-Sanchez et al. [25]
Mitochondria	Regulation of mitochondrial function and cell survival	Abramov et al. [26], ME Solesio et al. [27,28]
Nucleus/Nucleolus	Regulation of gene expression and nuclear processes.	Jimenez-Nunez et al. [29], Bru et al. [30], Kumble et al. [19]
Cytoplasm	General cellular signaling and metabolic regulation	Kumble et al. [19], Pavlov et al. [31].
Plasma membrane/ pericellular space	Extracellular signaling and modulation of coagulation and inflammation	Baker et al. [3], Rangaswamy et al. [32], Müller et al. [33].

**Table 3:** Evidence level and proposed roles of polyP in hematolymphoid tumors.

Evidence Level	Disease	Proposed Role of polyP	Supporting Evidence	Key Knowledge Gap
Direct evidence	Multiple myeloma	Intracellular nucleolar polyP; extracellular polyP-induced apoptosis	Published experimental studies [24,25]	Clinical significance
Preliminary evidence	Hodgkin lymphoma	Intracellular polyP detection	Unpublished observations (Salb, Santisteban-Espejo, and Ruiz, unpublished results)	Functional role
Indirect evidence	Acute leukemias	Proliferation by Ras/Akt signaling and/or metabolic regulation	Studies in other cancers [38,39]	Presence and function of polyP
Indirect evidence	Chronic leukemias	Mitochondrial regulation	General polyP biology	Disease-specific evidence
Hypothetical	Non-Hodgkin lymphoma	Microenvironment, thrombosis	Extrapolation	Experimental validation

## Data Availability

No new data were created or analyzed in this study. Data sharing is not applicable to this article.

## Supplementary Materials

The following supporting information can be downloaded at: https://www.mdpi.com/article/10.3390/biom16071036/s1, File S1: Detailed description of the search strategy used.

## Abbreviations

The following abbreviations are used in this manuscript:

PolyP	Inorganic Polyphosphate
P_i_	Inorganic Phosphate
ATP	Adenosine triphosphate
RNA	Ribonucleic acid
mTOR	Mechanistic target of rapamycin
PTP	Mitochondrial permeability transition pore
bFGF	basic Fibroblast Growth Factor
cHL	Classic Hodgkin Lymphoma
