# Supplementary material for: Inorganic Polyphosphate in Hematolymphoid Malignancies: Biological Rationale and Emerging Research Gaps
Source: Biomolecules. 2026 Jul 16;16(7):1036. doi: 10.3390/biom16071036 (PMC13406506; doi:10.3390/biom16071036)
Supplement: Supplementary file 1 [file biomolecules-16-01036-s001.zip › biomolecules-4382488-supplementary.pdf]

Supplementary information to

Search strategy

A narrative literature search was conducted using the PubMed/MEDLINE database, with no restriction on the publication date and a search cutoff of May 2026. The search strategy combined Medical Subject Headings (MeSH) and free-text terms related to polyphosphate (polyP), grouped into complementary thematic blocks addressing its biology and metabolism in mammals, hemostasis, blood coagulation, platelet activation, inflammation, cancer and solid tumors, mitochondrial metabolism, enzymes involved in polyP metabolism, and hematolymphoid malignancies. A targeted manual search was also performed to identify relevant resources not indexed in PubMed, including web-based tools, molecular visualization software, and the WHO Blue Books Online.

Articles were included that met at least one of the following criteria: studying polyP as the central object of research, regardless of the model organism, provided that the described mechanism was evolutionarily conserved or provided a structural or enzymatic basis transferable to human biology; characterizing enzymes involved in the synthesis or hydrolysis of polyP with functional demonstration of their activity; investigating the role of polyP in coagulation, hemostasis, platelet activation, or inflammation; analyzing the involvement of polyP or inorganic phosphate in the proliferation, apoptosis, metastasis, or metabolism of tumor cells, in both solid and hematological neoplasms; describing the role of polyP in the regulation of mitochondrial calcium, ATP synthesis, or cellular bioenergetics; constituting recent, high-quality reviews that synthesized the state of knowledge on polyP, used as a general context reference; or provide relevant biological, clinical or molecular data on hematolymphoid tumors that support the conceptual framework of the review. The search was restricted to original and review articles published in indexed and peer-reviewed journals, in English.

Studies focusing on polyphosphate in environmental microorganisms, plants, or bioremediation were excluded unless they provided a clear mechanistic link to mammalian biology. Studies evaluating polyphosphate exclusively as a biomaterial for tissue engineering, nanomedicine, or biomineralization without direct relevance to hematology or oncology were also excluded. Additional exclusion criteria comprised letters, editorials, comments, errata, and conference abstracts lacking substantial original data; duplicate publications or preprints when a peer-reviewed version was available; studies addressing inorganic phosphate rather than polymeric polyphosphate, except when included as a direct mechanistic comparator in the context of cancer; articles for which neither the full text nor a sufficiently informative abstract was available; studies on hematolymphoid malignancies without a direct or indirect association with polyphosphate metabolism, coagulation, mitochondrial function, or the tumor microenvironment; and publications in languages other than English for which no translation was available.

Study selection was performed in three sequential phases: title and abstract screening, thematic screening according to the eligibility criteria defined for each domain, and full-text assessment. Around 80 studies were reviewed, and 46 articles were retained. Resources not indexed in PubMed, including software, databases, and chapters from institutional classification systems, were identified through a targeted manual search and evaluated independently of the automated literature screening process.
